# Prognostic role of NF-YA splicing isoforms and Lamin A status in low grade endometrial cancer

**DOI:** 10.18632/oncotarget.13854

**Published:** 2016-12-10

**Authors:** Lucia Cicchillitti, Giacomo Corrado, Mariantonia Carosi, Malgorzata Ewa Dabrowska, Rossella Loria, Rita Falcioni, Giuseppe Cutillo, Giulia Piaggio, Enrico Vizza

**Affiliations:** ^1^ Department of Research, Advanced Diagnostics and Technological Innovation, Area of Translational Research, Regina Elena National Cancer Institute, Rome, Italy; ^2^ Department of Experimental Clinical Oncology, Gynecologic Oncology Unit, Regina Elena National Cancer Institute, Rome, Italy; ^3^ Department of Research, Advanced Diagnostics and Technological Innovation, Anatomy Pathology Unit, Regina Elena National Cancer Institute, Rome, Italy

**Keywords:** endometrial cancer, lamin A, NF-Y, estrogen receptor, miR-200 family

## Abstract

Although most cases of low grade (G1) endometrial cancer (EC) do not behave aggressively, in rare instances, can progress in a highly aggressive manner. In this study we analyzed formalin-fixed, paraffin-embedded (FFPE) EC tissues to find novel clinical and biological features to help diagnosis and treatment of G1 ECs s in order to better stratify patient risk of recurrence. A retrospective cohort of FFPE specimens from patients with EC (n=87) and benign tissue specimens (NE) from patients who underwent a hysterectomy to treat other benign disease (n = 13) were collected. Total RNA and proteins were extracted and analyzed, respectively, by quantitative PCR and western blotting. NF-YAs is expressed and lamin A is down-modulated in all high grade (G2 and G3) ECs. In G1 ECs, NF-YAs expression is heterogeneous being expressed only in a subset of these tumours. Interestingly, the G1 ECs that express NF-YAs display low levels of lamin A similar to those present in G2 and G3 ECs. Of note, this pattern of NF-YAs and lamin A expression correlates with tumor aggressiveness assessed by comparative analysis with estrogen receptor (ER) status and epithelial-mesenchymal transition (EMT) markers thus suggesting its potential role as biomarker of tumour aggressiveness in G1 EC. In all grade ECs, lamin A is strongly downmodulated, being its expression inversely correlated with tumor aggressiveness and its loss of expression. We identified NF-YAs and lamin A expression levels as novel potential biomarkers useful to identify G1 ECs patients with risk of recurrence.

## INTRODUCTION

Endometrial cancer (EC) is the most common genital tract malignancy and occurs in reproductive and postmenopausal women. Most EC cases are sporadic, with only 10% considered familiar [1]. In general, patients with EC have a good prognosis since early diagnosis is frequent and the disease has usually not spread beyond the uterus. However, the prognosis for recurrent or metastatic EC remains poor and in order to improve therapy it is important to understand the processes which inhibit and stimulate cancer progression. EC is classified as type I or type II based on histologic properties. Type I, also called the endometrioid type (EEC) because of its histologic similarity to the endometrium, accounts approximately 70–80% of sporadic EC. Most type I tumors occur in the setting of unopposed estrogen stimulation, leading to endometrial hyperplasia usually classified as grade 1, 2 or 3 (G1, G2 or G3) depending on their histological similarity to endometrium[1]. Unlike type I tumors, type II lesions are usually G3 and are not related to estrogen exposure or endometrial hyperplasia and include high risk malignancies, as serous papillary and clear cell carcinoma, generally [1]. Identification of patients with poor prognosis among the presumed low risk, G1 endometrioid cases represents a particular therapeutic challenge. Several prognostic factors, such as histological type, histological grade, surgical stage, pelvic lymph node involvement and myometrial invasion have been established [2, 3, 4], and [5]. Meanwhile, some biological molecules have been identified as prognostic markers in EC, such as KRAS, PTEN, EGFR, FGFR, P53, HER2, and estrogen receptors (ERs) [6], but more efforts are needed to identify novel biomarkers with a potential for a more systematic integration in clinical practice for individualized therapy in EC.

Nuclear transcription factor (NF-Y) is transcription factor that activates genes involved in growth promotion including cell cycle regulatory genes. Numerous findings highlight that NF-Y is involved in cancer [7], [8], [9] and [10]. NF-Y is composed of three different subunits: YA, YB and YC. Subunit NF-YA has two different isoforms, NF-YAl (long) and NF-YAs (short), resulting from alternative splicing. Previous studies demonstrated that NF-YAs is down-regulated, whereas NF-YAl is up-regulated, during differentiation of hESCs, mouse ES cells, and hematopoietic stem cells [11], [12] and [13]. Although mutations in NF-Y subunits have never been specifically identified in tumours, systematic examination of protein expression profiles indicates that NF-Y targets are upregulated in different types of cancer. Recently, informatics analysis and microarray expression profile studies conducted in various gynecological cancers, revealed that also in these tumors, several NF-Y target genes are upregulated [14].

Expression of ERs has been correlated with EC stage, histologic grade and survival [15]. Loss of ERs has been significantly associated with aggressive phenotype and poor survival in EC patients [16]. In particular, early stage, well differentiated ECs usually retain ERs expression, whereas advanced stage, poorly differentiated tumours often lack one or both receptors. In the human uterus, ER-α is the predominant subtype [17], and ER-β is supposed to play an i role by modulating ER-α function [18] and [19]. Recently, a link between hormone receptor status and epithelial-mesenchymal transition (EMT) has been recently proposed [20]. EMT enables epithelial tumor cells to acquire a like mesenchymal potential with increase motility and ability to extravasate and circulate. In EC, alteration of EMT markers, including several miRNAs, including miR200 family, have been identified in metastatic disease and associated with reduced survival [20].

Nuclear lamins are type V intermediate filament proteins that are critically for the structural properties of the nucleus and are involved in cell migration and differentiation [21], and [22]. A-type lamins, whose most represented isoforms are lamin A and C, are alternatively spliced products of the same gene. Loss of lamin A expression has been reported for several types of cancers [22], [23], and [24], recently also for also for EC [25].

In the present study, we assessed NF-YA isoforms and lamin A expression in several EC samples and identified them as novel potential prognostic EC biomarkers. We report for the first time that a specific NF-YA splicing isoform, NF-YAs, is associated with EC development. In our cohort of G1 EECs, NF-YAs was expressed in about 55% of samples, whereas it was detectable in all higher grade and non endometrioid (NEM) ECs. Also, our data indicate that an association between NF-YAs isoform and lower ER-α mRNA (ESR1) expression occurs. Interestingly, clustering of NF-YA isoforms in G1 EEC indicated an inverse association between NF-YAs and lamin A expression and a direct correlation with an increase of miR-200 levels.

Our findings suggest NF-YAs and lamin A as novel biomarkers with a potential for a more systematic integration in clinical practise for individualised therapy in EC, in particular in low grade malignancy.

## RESULTS

### NF-YA splicing isoforms are differentially expressed in EC tissues

**Figure 1:**
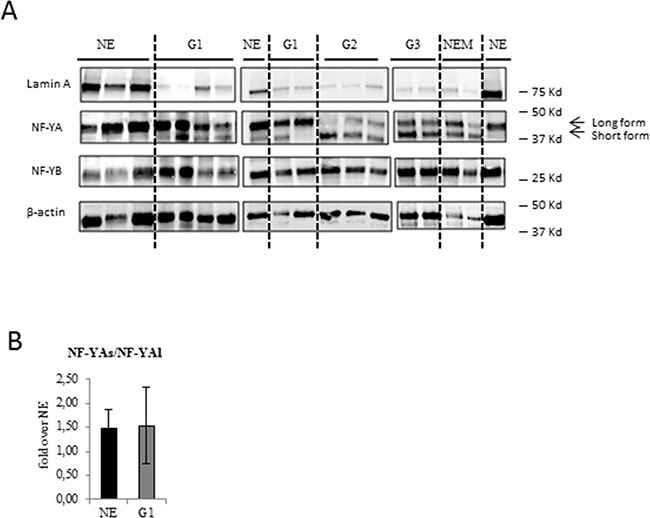
Analysis of NF-YA isoforms and lamin A protein expression in benign and EC tissues **A**. Representative immunoblottings of proteins extracted from benign (NE), low grade G1 (G1) and high grade endomentrial endometrioid cancer (G2-G3) and non endometrioid (NEM) EC FFPE tissues with anti NF-YA, anti-NF-YB, anti-Lamin A antibodies. The dashed lines separate the different groups. Anti-β actin was used as loading control. **B**. Average expression of the ratio NF-YAs to NF-YAl mRNA expression examined by qRT-PCR±SD in G1 EEC tissues. mRNA expression was normalized for 18S rRNA levels. The error bars indicate the standard error.

We analysed the expression of NF-YA and NF-YB subunits in several EC FFPE specimens, whose clinic-pathological features are described in Table 1, and compared the protein expression level of the two subunits in EC and NE FFPE specimens (Figure 1A).

**Table 1:** Clinicopathological features of 87 ECs

Clinico-pathological features	Total EECs	G1 EECs	G2-G3 EECs	NEM
**No of cases**	78	29	49	9
**Median Age (years)**	63 (range 42-88)	58 (range 42-76)	66 (range 43-88)	65 (range 50-74)
**BMI > 30**	14 (17.9%)	8 (27.5%)	6 (12.2%)	7 (77.7%)
**MI > 50%**	33 (42.3%)	5 (17.2%)	28 (57.1%)	7 (77.7%)
**Lymph node metastases**	4 (5.1%)	0 (0.0%)	4 (8.1%)	2 (22.2%)
**FIGO Stage**				
I and II	40 (51.2%)	29 (100%)	21 (42.8%)	6 (66.6%)
III and IV	28 (35.9%)	0 (0.0%)	28 (57.1%)	3 (33.3%)
**Site of recurrence**				
Local	17 (21.8%)	0 (0.0%)	17 (34.7%)	0 (0.0 %)
Distant	10 (12.8%)	2 (6.9%)	8 (16.3%)	0 (0.0 %)
Multifocal	14 (17.9%)	0 (0.0%)	14 (28.6%)	3 (33.3%)
**RT**	29 (37.1%)	3 (10.3%)	26 (53.0%)	4 (44.4%)
**CHT**	24 (30,7%)	0 (0.0%)	15 (30.6%)	9 (100%)

RT= adjuvant radioteraphy; CHT=adjuvant chemoteraphy; BMI= body mass index;

MI= myometrial infiltration.

We did not detect any significant modulations of NF-YA and NF-YB protein levels in ECs compared to NE tissues but, very interestingly, we observed a different and specific electrophoretic profile of NF-YA splicing isoforms. We found that in NE tissues the long isoform of NF-YA (NF-YAl) was the main form expressed, whereas the short isoform (NF-YAs) was mostly undetectable. Interestingly, NF-YAs was clearly detectable in EC tissues (Figure 1A), suggesting its specific association with a tumour phenotype. NF-YAs was expressed in all higher grades, G2 and G3 EEC, and NEM tissues, whereas it was detected only in 55% of our G1 samples (G1). This result suggests that NF-YAs could represent a marker of EC and, also, an indicator of tumour aggressiveness. Assessment of the ratio of mRNA expression of NF-YAs and NF-YAl (NF-YAs/NF-YAl) by qRT-PCR using primers designed to specifically amplify only NF-YAl or NF-YAs mRNA was very similar in NE and G1 EEC tissues (Figure 1B), thus indicating that protein levels of NF-YA isoforms very likely depend on post-transcriptional mechanisms.

### NF-YAs detection in G1 EEC tissues correlates with decreased ERs mRNA expression

**Figure 2:**
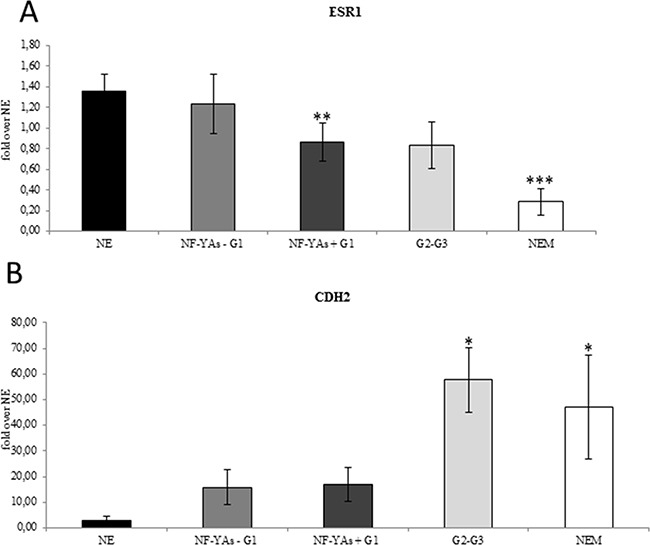
ESR1 decreased mRNA levels are associated with increase CDH2 expression Average of expression of the ESR-1 mRNA **A**., and of CDH2 **B**. mRNA in EC tissues examined by qRT-PCR±SD. mRNA expression was normalized for 18S rRNA levels as endogenous control. Statistical significance: *P≤0.05, **P≤0.01, ***P≤0.001 vs NE. The error bars indicate the standard error.

We first analysed ERs status to evaluate its prognostic values in our patient cohort (Table 2). In G1 EEC, 27.5% and 31% of tissues were found to express low ER-α (ESR1) and ER-β (ESR2) mRNA levels, respectively. A large down-modulation of ESR1 and ESR2 mRNA, in 61.2% and 77.5% of high grade EC and 77.7% and 88.8% NEM samples respectively, was observed (Table 2), thus indicating that reduction of ERs level is related with advanced stage of EC. It is worth noting that ESR2 mRNA expression levels were very variable in our cohort of specimens. Based on these evidences, we focused on ESR1 status to correlate its expression level in EC subtypes. Results displayed a correlation of reduction of ESR1 expression with an aggressive clinicopathologic phenotype (Figure 2A). Then, we focused our attention on ESR-1 mRNA expression and the expression of NF-YA isoforms in G1 EECs, where a heterogeneous NF-YA expression of the two splicing isoforms was observed. We observed an association between the presence of NF-YAs and lower ESR1 mRNA levels (Figure 2A and Table 2), thus indicating a possible involvement of NF-YAs in EC aggressiveness. It is worth to note that ESR1 mRNA levels in NF-YAs positive tissues were very similar to that of higher grades (G2 and G3), whereas in NF-YAs negative (NF-YAs-) specimens were comparable to those of NE tissues (Figure 2 and Table 2). These results strongly support a correlation between ERs status and NF-YAs expression.

**Table 2:** EC histologic grade in relation to low levels of LMNA, ESR1, ESR2 expression, and E/N index

*ERs, LMNA and E/N mRNA expression in EC*	Low ESR1 n (%)	Low ESR2 n (%)	Low LMNA n (%)	Low E/N n (%)
NE (n=13)	0 (0%)	6 (46.1%)	0 (0%)	2 (15.3%)
G1 EEC (n=29)	8 (27.5%)	9 (31%)	20 (68.9%)	9 (31%)
G1 EEC NF-YAs- (n=13)	3 (23%)	3 (23%)	5 (38.5%)	4 (30.7%)
G1 EEC NF-YAs+ (n=16)	5 (31.2%)	6 (37.5%)	15 (93.7%)	5 (31.2%)
G2-G3 EEC (n=49)	30 (61.2%)	38 (77.5%)	43 (87.7%)	25 (52%)
NEM (n=9)	7 (77.7 %)	9 (88.8%)	7 (77.7%)	4 (44.4%)

EC histologic grade in relation to low levels of LMNA, ESR1, ESR2 expression, and E/N (CDH1/CDH2) index. LMNA, ESR1, ESR2, CDH1 and CDH2 mRNA were examined by qRT-PCR. Low level = mRNA expression in EC over benign samples (fold over NE) ≤ 0,5.

### Upregulation of miR-200 family inversely correlates with ZEBs expression in ECs and is related to NF-YAs expression in G1 EEC

**Figure 3:**
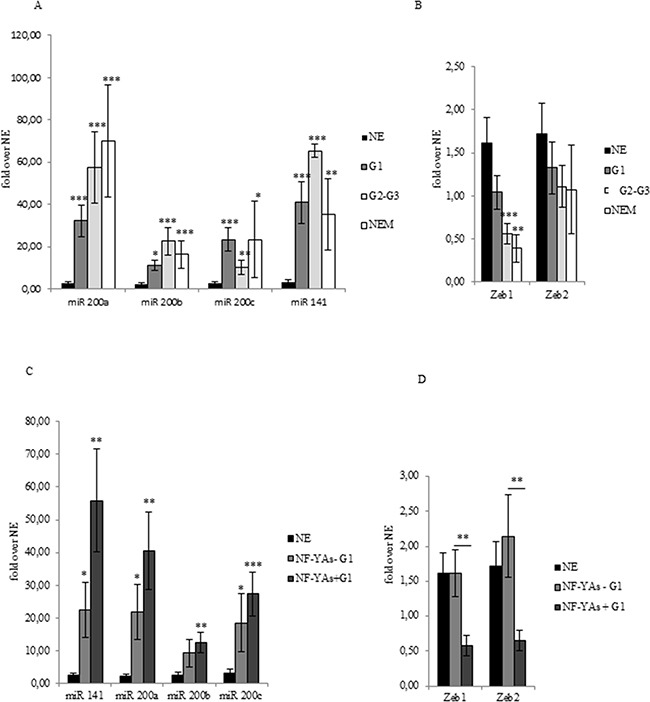
miR-200s and ZEBs expression in EC FFPE tissues Average of miR-200 family members (miR-200a, miR-200b, miR-200c, and miR-141) expression **A**, of ZEB1 and ZEB2 **B**, mRNA in benign (NE), G1 EEC, G2-G3 EEC, and NEM FFPE tissues. Average of miR-200 family members (miR-200a, miR-200b, miR-200c, and miR-141) expression **C**, and of ZEB1 and ZEB2 **D**, mRNA in benign (NE), NF-YAs positive (NF-YA+) and negative (NF-YAs-) G1 subsets of EEC FFPE tissues. Analysis was performed by qRT-PCR±SD. miRNA expression was normalized using small nuclear RNA U6 as endogenous control. mRNA expression was normalized for 18S rRNA levels as endogenous control. Statistical significance: *P≤0.05, **P≤0.01, ***P≤0.001, The error bars indicate the standard error.

Lack of ER-α has been recently associated with EMT [26]. The miR-200 family has been extensively studied with respect its role in regulating genes of EMT. It is worth noting that upregulation of miR-200a and ERs loss have been associated with poor prognosis [29]. Analysis of the qRT-PCR data showed that all members of the miR-200 family analysed (miR-200a, miR- 200b, miR-200c, and miR-141) were up-regulated in all stages of EC compared to NE tissues (Figure 3A), which confirms results reported in previous studies [27, 28] and [29]. In order to validate the activity of miR-200s in EC, we also analysed the expression level of ZEB1 and ZEB2 (Figure 3B), well established as direct targets of miR-200 family. In EC, levels of ZEB1 and ZEB2 were lower and further decreased in NEM, thus supporting the hypothesis of an augmented translational activity of miR-200 family members in more aggressive phenotype. Then, we focused our analysis in our subset of G1 samples. Interestingly, we observed a consistent increase of miR-200 family expression (Figure 3C) inversely related to ZEBs mRNA levels in NF-YAs positive compared with negative NF-YAs G1 EEC samples (Figure 3D), thus supporting the hypothesis of a correlation between NF-YAs expression and tumour aggressiveness. Finally, to better characterize the possible EMT involvement in G1 EEC, we assessed N-cadherin mRNA expression (*CDH2*), a mesenchymal marker, by qRT-PCR, and the ratio of E-cadherin to N-cadherin (E/N), an index of differentiated phenotype. Levels of *CDH2* were augmented in all EC tissues and a further significant increase was observed in more aggressive phenotypes (Figure 2B). Moreover, as shown in Table 2, we found that 31%, 52%, and 44% of cases displayed low E/N index in G1, G2-G3 and NEM, respectively, thus indicating a trend towards EMT in more aggressive ECs. Analysis on our G1 tissues displayed the lack of any modulation of both *CDH2* mRNA levels and E/N ratio in NF-YAs positive compared with NF-YAs negative (Figure 2B and Table 2), suggesting that *CDH1* and *CDH2* expression is not very likely associated with differential expression of NF-YA isoforms in G1 EEC.

### Decreased lamin A levels are associated with EC progression and aggressiveness and NF-YAs expression in G1 EEC

**Figure 4:**
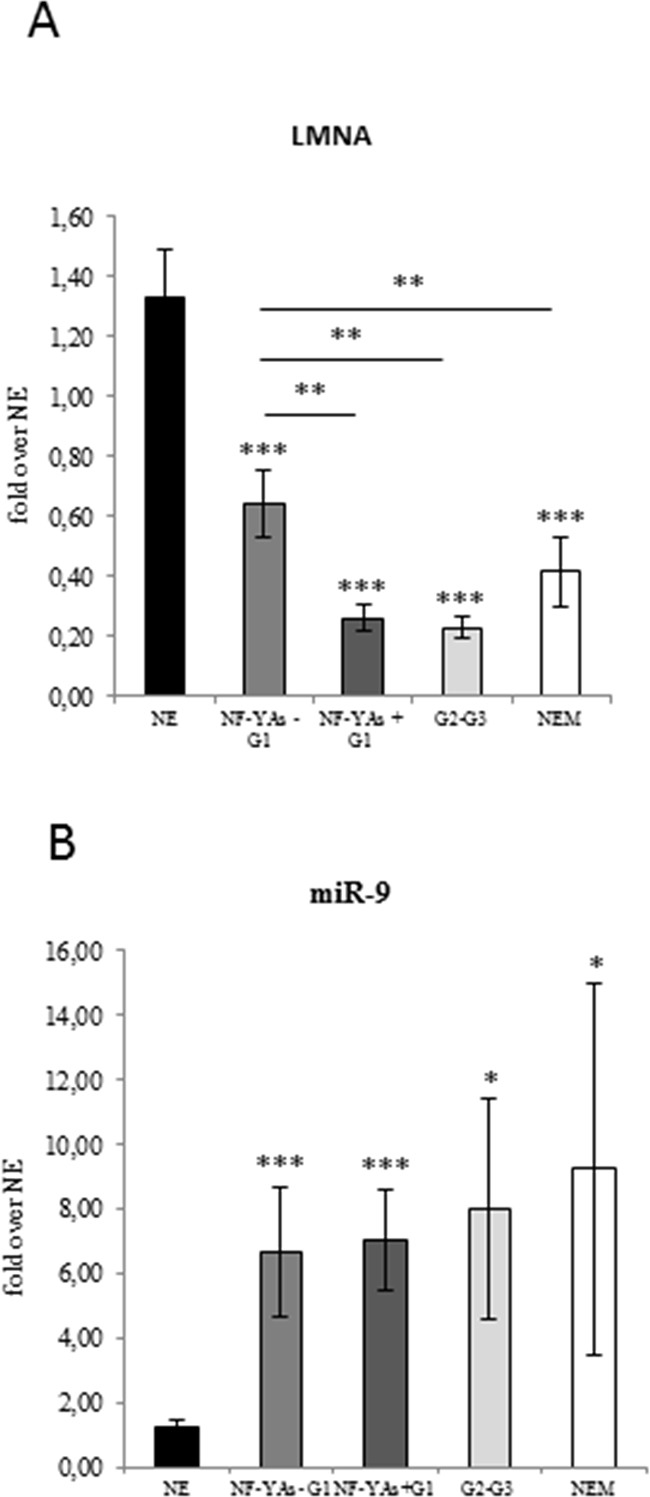
Evaluation of lamin A and mir-9 expression in ECs Average of expression of LMNA mRNA **A**. and miR-9 **B**. in benign (NE), in the two subsets of FFPE G1 EEC tissues (NF-YAs- and NF-YAs+) G1 EEC, G2-G3 EEC, and NEM FFPE tissues examined by qRT-PCR±SD. mRNA expression was normalized for 18S rRNA levels. miRNA expression was normalized using small nuclear RNA U6 as endogenous control. Statistical significance: *P≤0.05, **P≤0.01, ***P≤0.001. The error bars indicate the standard error.

Lamin A has been reported to be a direct target of miR-9 and that upregulation of miR-9 expression occurs in EC [30]. We assessed lamin A protein (Figure 1A) and mRNA expression (Figure 4A), and miR-9 levels (Figure 4B) in our cohort of EC tissues. Results displayed loss of lamin A expression in EC and that reduced *lamin A* mRNA levels were associated with overexpression of miR-9 (Figure 4B). Interestingly, data obtained by comparing *lamin A* mRNA levels in the two subgroups of G1 EEC tissues indicated that *lamin A* reduction was significantly higher (*P* ≤ .01) in NF-YAs positive with respect to NF-YAs negative samples (Figure 4A). Moreover, levels of lamin A in NF-YAs positive tissues were comparable to that of high grade EEC. These evidences further support the potential role of NF-YAs and lamin A as molecular indicator of tumour aggressiveness in early stage EC.

## DISCUSSION

A recent study based on both informatics analysis and experimental evidence identified NF-Y as one of the key components of the transcription deregulation in gynecological cancer [14]. In agreement, here we identified a specific splicing isoform of the regulatory subunit of NF-Y, NF-YA, as a new potential indicator of aggressiveness in low grade G1 EEC. Indeed, although most cases of G1 EEC do not behave aggressively, in rare instances, even low-grade, well-differentiated endometrial adenocarcinomas can progress in a highly aggressive manner. We observed that NF-YA short isoform (NF-YAs) was undetectable in benign tissues, while NF-YA long isoform (NF-YAl) is always expressed, whereas NF-YAs was consistently expressed in high grade G2/G3 EEC and in NEM subtypes. Interestingly, in low grade G1 EECs a heterogeneous expression of NF-YAs was observed with some samples expressing exclusively the NF-YAl and others samples expressing both isoforms. Based on this observation we are now able to stratify low grade G1 EEC in two subgroups: one expressing only NF-YAl, NF-YAs negative (NF-YAs-), and another expressing both isoforms, NF-YAs positive (NF-YAs+). The exclusive presence of NF-YAl isoform in benign tissues suggests that it may represent a marker of differentiation and that the presence of NF-YAs may be linked with an increase of a pool of poorly differentiated cells in tumors tissues.

To better characterize the significance of the heterogeneous expression of NF-YA isoforms in G1 EEC, we analyzed the estrogen receptor (ER) status. It has been well documented that higher level of ERs are significantly associated with good prognosis, and that early stage-well differentiated EC usually retain their expression, whereas poorly differentiated tumors often lack one or both of these receptors [18] and [26]. In the current study we first confirmed this correlation, than by clustering of samples expressing and not expressing NF-YAs in G1 EEC we highlight a strong correlation between loss of ERs and presence of NF-YAs.

A recent study has reported that lack of ER-α correlates with epithelial mesenchymal transition and metastasis [26]. In agreement, ERs regulate transcription of miR-200 family members, a family of miRNAs that are known to maintain an epithelial phenotype in several cancers among which in low grade EEC [31], [32], [33], [34] and [35]. In G1 EEC we observed an upregulation of miR-200 family members. A significant increase of N-cadherin mRNA and a lower E-cadherin/N-cadherin index was observed in more aggressive EC tissues, thus confirming the association of high EMT signature with tumor aggressiveness and poor prognosis. However, our analysis does not revealed a correlation between N-Cadherin, E-Cadherin and NF-YAs expression in low grade G1 EEC.

A retrospective analysis in our cohort of low grade G1 EEC revealed that the two patients developing liver and lung recurrences both belonged to the NF- YAs positive subgroup. Considering the relative rarity of distant metastasis in G1 EEC (< 5%), and our low number of cases, further studies are needed to evaluate the possible correlation between NF-YAs expression and risk of recurrence.

Lamin A expression frequently correlates with cancer subtypes and cancer aggressiveness, proliferative capacity and differentiation state [22], [23], [24] and [25]. Recently, we reported that alteration of lamin A levels may be use as prognostic biomarker in EC and a correlation of its decreased expression levels with an increased myometrial infiltration in early stages of EEC [25]. Very interestingly, we observed an inverse correlation between NF-YAs and lamin A expression, thus further supporting the hypothesis of a possible involvement of NF-YAs in tumor differentiation and aggressiveness. In agreement with this, we have recently characterized a role for lamin A in counteracting NF-Y transcriptional activity in cancer cells [36]. Thus, we hypothesizes that concomitant expression of NF-YAs and lamin A down-modulation may be associated with an increased proliferation rate and a more invasive phenotype of cancer cells.

Further investigations are needed to confirm and better characterize our findings, considering also the new genomic characterisation of EC recently presented by the Cancer Genome Atlas Research Network (TCGA) [37].

In conclusion, our data highlight that a combination of NF-YAs, lamin A and ERs expression could be a novel potential predictive signature for new stratification approaches in low grade G1 ECs.

## MATERIAL AND METHODS

### Patient cohort

A retrospective cohort of formalin-fixed, paraffin-embedded (FFPE) specimens from patients with EC (n=87) and NE specimens (FFPE) from patients who underwent a hysterectomy to treat other benign disease (n = 13) were collected. According with the histologic grade, we analysed 29 low grade (G1), 49 high grade (G2/G3) ECs and 9 non endometrioid tissues (NEM). 79,6% of high grade (G2/G3) patients developed metastasis (Table 1). Biopsies were sampled for primary tumors in hysterectomy specimens.

### RNA extraction and RT-PCR

Total RNA derived from FFPE tissues was extracted using the PureLink™ FFPE Total RNA Isolation Kit (Invitrogen) following the manufacturer's instructions and reverse-transcribed using PrimeScript RT reagent kit (Takara). The quality of the total RNA was measured using a NanoDrop 2000 spectrophotometer (Thermo Fisher Scientific, Wilmington DE, USA). Quantitative PCR (qPCR) was performed using SYBR Select (Applied Biosystems) on an ABI Prism 7500 apparatus (Applied Biosystems). mRNA expression was normalized for 18S rRNA levels. Relative mRNA expression was calculated using the comparative Ct method (2−ΔΔCt). Primers used are listed in Table 3.

**Table 3:** PCR primers for mRNA quantification

NF-YAs fw	ACAGATTCAGCAGCAGGTCC
NF-YAs rv	ATGGGTTGGCCAGTTGATGT
NF-YAl fw	CAGGGTGGTGTCACTGCTG
NF-YAl rv	TACCTGGAGGGTCTGGACTT
LMNA fw	GGACAATCTGGTCACCCGC
LMNA rv	TGGCAGGTCCCAGATTACATG
ESR1 fw	TACTGACCAACCTGGCAGACAG
ESR1 rv	TGGACCTGATCATGGAGGGT
ESR2 fw	AGTTGGCCGACAAGGAGTTG
ESR2 rv	CGCACTTGGTCGAACAGG
ZEB1 fw	AACCACCCTTGAAAGTGATCCA
ZEB1 rv	CTTGTCTTTCATCCTGATTTCCATT
ZEB2 fw	CAAAGGAGAAAGTACCAGCGGA
ZEB2 rv	CATCAAGCAATTCTCCTGAAATCC
CDH1 fw	CCCACCACGTACAAGGGTC
CDH1 rv	ATGCCATCGTTGTTCACTGGA
CDH2 fw	AGAAGAAGACCAGGACTATGACTTGAG
CDH2 rv	ACAGTGTCAGGCTGCTGCAG
18S rRNA fw	CCTGGATACCGCAGCTAGGA
18S rRNA rv	GCGGCGCAATACGAATGCCCC

### MicroRNA analysis

Reverse transcription and qRT-PCR amplification were performed in two steps. In the first reverse transcription step, 10 ng of RNA was used in reactions with specific stem-loop RT primer for miR-200a, miR-200b, miR-200c, miR-141, and miR-9 and endogenous control primer for small nuclear RNA U6. Reaction was performed with TaqMan MicroRNA Reverse Transcription Kit, according to the manufacturer's protocol (Applied Biosystems, Foster City, CA). In the second step, cDNA samples were amplified in Real Time PCR instrument 7500 (Applied Biosystems) with the specific TaqMan miR-200a, miR-141, and miR-205 assay and small nuclear RNA U6 as endogenous control. The relative quantity (RQ) of each miRNA was calculated by the comparative CT (2-ΔΔCT) method, in which ΔΔCT was calculated as follows: ΔΔCT = (CTmiR-of-interest - CTU6)cancer - (CTmiR-of-interest - CTU6)benign.

### Immunoblotting

The paraffin from thin sections of FFPE specimens was melted at 72°C for 20 minutes using heat in the presence of a specially designed Melting Buffer contained in the PureLink™ FFPE Isolation Kit used for RNA extraction (Invitrogen). Tissues were then separated from the melted paraffin by centrifugation. Proteins were extracted in a high pH lysis buffer (20 mM Tris HCl pH 9.0, 0.2 M Glycine, 2% (w/v) SDS). The samples were first incubated on ice for 5 min, and mixed by vortexing, then boiled at 100°C for 20 min followed by an l hour incubation at 80° C for 2 hours. After extraction, any remaining unsolubilized material was pelleted at 14000 × g for 20 minutes, and protein concentration of total protein extracted was determined by the BCA Protein Assay (Pierce Chemicals Co., Rockford, IL, USA). The Pierce BCA Protein Assay is a detergent compatible formulation and the protein standards were prepared using the same lysis buffer as the samples. Proteins were resolved by SDS-PAGE and electrotransferred to nitrocellulose. Each membrane was blocked with 5% non-fat dry milk in Tris buffered saline-Tween-20 (TBST) for 1 hour at room temperature and subsequently incubated with primary antibody for 16 hours at 4˚C. The following antibodies were used: anti-NF-YB monoclonal (Santa Cruz), anti-NF-YA monoclonal (Santa Cruz), anti-Lamin A (Santa Cruz), and anti-β actin (sigma-aldrich). Immunoreactivity was detected by sequential incubation with HRP-conjugated secondary antibody.

### Statistical analysis

Data were reported as mean and standard deviation. In all experiments, comparisons of results from qRT-PCR between two groups were based on Student's t-test and one-way analysis of variance (ANOVA). P≤0.05 was deemed to be significant variation.

## Abbreviations

EC: Endometrial cancer
EEC: Endometrioid endometrial adenocarcinoma
NEM: Non endometrioid endometrial adenocarcinoma
NE: Benign tissue
FFPE: Formalin-fixed, paraffin-embedded
NF-YAs: NF-YA short form
NF-YAl: NF-YA long form
ER: Estrogen receptor
EMT: Epithelial-mesenchymal transition
